# FMRpolyG accumulates in FMR1 premutation granulosa cells

**DOI:** 10.1186/s13048-020-00623-w

**Published:** 2020-02-26

**Authors:** M. Friedman-Gohas, S. E. Elizur, O. Dratviman-Storobinsky, A. Aizer, J. Haas, H. Raanani, R. Orvieto, Y. Cohen

**Affiliations:** 1 Sackler Faculty of Medicine, Tel-Aviv University, Tel Aviv, Israel; 2 IVF Unit, Chaim Sheba Medical Centre, Tel-Hashomer, 52621 Ramat-Gan, Israel

**Keywords:** FMRpolyG, FMR1 premutation carriers, RAN translation, FXPOI, COV434

## Abstract

**Background:**

Fragile X premutation (Amplification of CGG number 55–200) is associated with increased risk for fragile X-Associated Premature Ovarian Insufficiency (FXPOI) in females and fragile X-associated tremor/ataxia syndrome (FXTAS) predominantly in males. Recently, it has been shown that CGG repeats trigger repeat associated non-AUG initiated translation (RAN) of a cryptic polyglycine-containing protein, FMRpolyG. This protein accumulates in ubiquitin-positive inclusions in neuronal brain cells of FXTAS patients and may lead to protein-mediated neurodegeneration. FMRpolyG inclusions were also found in ovary stromal cells of a FXPOI patient. The role of FMRpolyG expression has not been thoroughly examined in folliculogenesis related cells. The main goal of this study is to evaluate whether FMRpolyG accumulates in mural granulosa cells of FMR1 premutation carriers. Following FMRpolyG detection, we aim to examine premutation transfected COV434 as a suitable model used to identify RAN translation functions in FXPOI pathogenesis.

**Results:**

FMRpolyG and ubiquitin immunostained mural granulosa cells from six FMR1 premutation carriers demonstrated FMRpolyG aggregates. However, co-localization of FMRpolyG and ubiquitin appeared to vary within the FMR1 premutation carriers’ group as three exhibited partial ubiquitin and FMRpolyG double staining and three premutation carriers demonstrated FMRpolyG single staining. None of the granulosa cells from the five control women expressed FMRpolyG. Additionally, human ovarian granulosa tumor, COV434, were transfected with two plasmids; both expressing 99CGG repeats but only one enables FMRpolyG expression. Like in granulosa cells from FMR1 premutation carriers, FMRpolyG aggregates were found only in COV434 transfected with expended CGG repeats and the ability to express FMRpolyG.

**Conclusions:**

Corresponding with previous studies in FXTAS, we demonstrated accumulation of FMRpolyG in mural granulosa cells of FMR1 premutation carriers. We also suggest that following further investigation, the premutation transfected COV434 might be an appropriate model for RAN translation studies. Detecting FMRpolyG accumulation in folliculogenesis related cells supports previous observations and imply a possible common protein-mediated toxic mechanism for both FXPOI and FXTAS.

## Introduction

Fragile X Syndrome (FXS) is the most common form of inherited mental retardation caused by a trinucleotide repeat expansion (CGG) in the 5′-untranslated region of the fragile X mental retardation 1 (FMR1) gene located at Xq27.3. Patients with fragile X–related mental retardation carry the full CGG-repeat expansions mutation (> 200 repeats), generally accompanied by hypermethylation of the promoter region, along with the consequent transcriptional silencing of the FMR1 gene and absence of the encoded FMR1 protein (FMRP) [1].

Expansion of the CGG triplet number above the normal range (*n* > 55) towards the premutation status (*n* = 55–200) is associated with increased risk for fragile X-associated premature ovarian insufficiency (FXPOI) in females [2, 3], and fragile X-associated tremor/ ataxia syndrome (FXTAS). FXTAS is a late-onset neurodegenerative disease with symptoms in 50% among males over 50y and 8% among women [4]. FXTAS leads to a progressive degenerative movement disorder characterized by kinetic tremor, cerebellar gait ataxia, parkinsonism, and cognitive decline [5, 6]. FXPOI is clinically significant and defined as FMR1 premutation carriers having reduced fertility. Symptoms of FXPOI include menopause before the age of 40 years, ovarian dysfunction, and decreased fertility as evident by abnormal ovarian reserve biomarkers and a reduced ovarian response to controlled ovarian hyperstimulation (COH). These symptoms result in higher gonadotropin dosages and fewer embryos [7–13]. 20% of FMR1 premutation female carriers may suffer from premature ovarian insufficiency (POI) compared to only 1% of the general population [8]. Both male as female FMR1 premutation carriers have increased *FMR1* transcript levels and a normal or slightly reduced levels of the FMR1 protein (FMRP) [14].

The pathobiology of FXPOI is unclear, whereas the understanding of the molecular mechanism of FXTAS is advancing. The RNA gain-of-function mechanism resulting in RNA toxicity and a non-canonical protein translation creating a cryptic protein, FMRpolyG, are two major mechanisms of FXTAS that have been described in the literature [15, 16]. Which pathological mechanism drives FXPOI pathogenesis remains a crucial question.

The RNA gain-of-function mechanism has been best established in Myotonic Dystrophy Type I, where a CUG repeat expansion in the 3′ UTR of *DMPK* binds to and sequesters the Muscleblind (MBNL) family of RNA-splicing factors [17–19]. In FXTAS, the RNA gain-of-function mechanism has been demonstrated by numerous groups. They have suggested that the CGG repeat can provoke RNA toxicity, presumably by sequestering specific RNA-binding proteins, including hnRNP A2, Pur α, SAM-68 and the miRNA biogenesis complex Drosha/ DiGeorge critical region 8 (DGCR8), that are critical for normal cell function. These proteins could undergo a loss of function [15, 16, 20–23]. However, the role of these interactions in the disease pathogenesis remain incomplete.

The second major mechanism is related to the accumulation of toxic FMRpolyG protein in several tissues of FXTAS patients. It has been recently shown that the CGG repeats expansion triggers repeat associated non-AUG initiated (RAN) translation of a cryptic polyglycine-containing protein, FMRpolyG. FMRpolyG accumulates in ubiquitin-positive neuronal inclusions, a pathologic hallmark of protein-mediated neurodegeneration. Several studies demonstrated that FMRpolyG accumulates in ubiquitin-positive inclusions in Drosophila, cell culture, mouse disease models and FXTAS patient brains [16, 24, 25]. Buijsen et al. revealed co-localization of ubiquitin and FMRpolyG in FXTAS patient and in ovary stromal cells from a FXPOI 42 years old patient but not in folliclogenesis related cells [26].

Sellier and Todd found that translation of expanded CGG repeats occurs predominantly in the glycine frame through initiation at a near-cognate ACG codon located upstream of the expanded CGG repeats. Transgenic mice expressing both CGG RNA repeats and the polyglycine protein (99CGG with FMRpolyG mouse), but not mice expressing only the mutant RNA containing expanded CGG repeats (CGG without FMRpolyG mouse), exhibit inclusion formation, motor phenotypes, and reduced lifespan [16]. Therefore, they have concluded that translation of expanded CGG repeats into FMRpolyG may have a key role in contribution to FXTAS.

Prompted by these aforementioned observations we aim to explore whether the molecular mechanism shows similarities between the pathogenesis of FXPOI and FXTAS. In this study, we aimed to examine whether FMRpolyG is expressed in mural granulosa cells from FMR1 premutation carriers and evaluate premutation transfected COV434 as a disease model, to examine FMRpolyG presence and possible role in the pathogenesis of FXPOI.

## Material and methods

This study was approved by the Institutional Ethical Review Board of Sheba Medical Center, Israel. All patients that were included in this study signed a written informed consent. Helsinki no. 8707–11-SMC and 6140–19-SMC. In Israel, as part of a national prenatal screening program, all women who wish to conceive are advised to determine their FMR1 CGG carrier status. All FMR1 premutation carriers (55–200 CGG repeats) are further referred to a genetic consultation to consider in vitro fertilization (IVF) and pre-implantation genetic diagnosis (PGD) to avoid the risk of CGG expansion in offspring. Therefore, the CGG repeat status is known for all women undergoing IVF in our center (either FMR1 premutation carriers (55–200 CGG repeats) or normal (< 55 CGG repeats).

The study population consisted of five FMR1 premutation carriers referred to our IVF unit for IVF-PGD treatment who reached the ovum pick-up (OPU) stage. The control group consists of five patients matched by age, with less than 55 CGG repeats, undergoing IVF-ICSI for male factor infertility in the same study period. The treating physician was responsible for selecting of the type of COH protocol to be used. In all protocols, gonadotropins were administered in variable doses, depending on patient age and/or ovarian responsiveness in previous cycles, and further adjusted according to serum estradiol (E_2_) levels and vaginal ultrasound measurements of follicular diameter obtained every 2 or 3 days. Thirty-four to 36 h following HCG injection, oocytes were aspirated using the ultrasound guided transvaginal route and the pooled follicular fluids containing mural granulosa-cells were collected from each patient.

### Mural granulosa cells culture

Following oocyte retrieval, mural granulosa cells from follicular fluid were washed with 1x PBS to remove the residual blood. The cells were re-suspended in culture medium, Dulbecco’s Minimum Essential Medium (DMEM; Biological Industries) supplemented with 5% fetal bovine serum (Biological Industries), 1% l-Glutamine (Biological Industries) and 1% penicillin/streptomycin (Biological Industries) onto sterilized coverslips lining in wells of a plastic 24 well tissue culture plates. The cells were incubated in a humidified atmosphere of 5% CO2 in air at 37 °C. The granulosa cells were left to attach for 24 h. Then the culture medium was replaced every 24 h for 4 additional days.

As anti Müllerian hormone (AMH) is secreted by follicles and granulosa cells [27], we performed anti-AMH immunostaining to confirm the cells obtained from follicular fluid are granulosa cells. Was performed as described in “Cells fixation and Immunofluorescence” (Fig. 2S).

### RNA isolation and real-time quantitative PCR

Total RNA isolation was performed using TRIzol reagent (Life Technologies, Invitrogen, RHENIUM Ltd. Modi’in, Israel) according to the manufacturer’s protocol and then reverse transcribed into complementary deoxyribonucleic acid (cDNA) using random hexamers (Amersham Biosciences, Buckinghamshire, UK) and Moloney murine leukaemia virus reverse transcriptase (Promega, Madison, WI, USA).

Real-time quantitative PCR was performed using the StepOnePlus™ System (Applied Biosystems). The mRNA levels of genes were measured by SYBR Green Fast (Invitrogen, Rhenium Ltd. Modi’in, Israel) according to the manufacturer’s instructions. The human beta-actin was used as a control housekeeping gene. Melting curve analysis was performed to confirm amplification of specific transcripts. The expression levels of transcripts were calculated by the relative quantification (DDCt) study method by using SDS software (Applied Biosystems).

Primer sequences used for amplifications were as follows:

- h-FMR1_F: AAC AAA GGA CAG CAT CGC TAA TG
- h-FMR1_R: CAA ACG CAA CTG GTC TAC TTC CT
- h-b-ACTIN_F: CCT GGA CTT CGA GCA AGA GA
- h-b-ACTIN_R: CAG CGG AAC CGC TCA TTG CCA ATG G

### Statistical analysis

The statistical analysis was performed using 2 tailed unpaired Student t test. The differences among groups were considered significant when *p*-value was under 0.05.

### Cells fixation and immunofluorescence

Brain sections were de-paraffinized using Xylene and Ethanol and antigen retrieval was performed using 10% formic acid.

Transfected COV434 were grown on coverslips for 72 h following transfection and human mural granulosa cells were grown 4 days on coverslips with medium replacement. The cells were then washed with PBS and permeabilized in 0.5% Triton X-100 in 4% PFA for 15 min. Cells were then fixed in 4% PFA for 20 min.

Following blocking in 5% bovine serum albumin (BSA) to avoid nonspecific binding, brain sections and granulosa cells were immunostained over night at 4 °C with a primary antibody. To study the distribution of FMRpolyG protein, we used two novel mouse monoclonal antibodies directed against the N- and C-terminus of FMRpolyG (8FM (1:200) and 9FM (1:200) respectively), a kind gift from Dr. Charlet-Berguerand [16, 26, 28]. Immunofluorescent double staining using antibodies against ubiquitin (Abcam, Cambridge, England. Ab7254; 1:200, mouse monoclonal) and FMRpolyG was used to study co-localization of ubiquitin- and FMRpolyG-positive inclusions. Anti-AMH Rabbit anti-human (Abcam, Cambridge, England. Ab229212; 1:200). After subsequent washes with 1xPBS, the cells were incubated for 1 h with the following secondary fluorescent antibodies: Goat anti-Rabbit alexa flour 568 (Abcam, Cambridge, England. Ab175471; 1:200), Goat anti-mouse, alexa flour 488 (Abcam, Cambridge, England. Ab150113; 1:200) and Goat anti-mouse, Cy3 (Abcam, Cambridge, England. Ab97035; 1:200). The cells were then nuclear counterstained with DAPI Fluoromount-G® (SouthernBiotech, Inc., Birmingham, AL, USA). Images were obtained using a confocal microscope (Zeiss LSM710, Oberkochen, Germany).

### Western blot analysis

Mural granulosa cells from follicular fluid were homogenized in a RIPA buffer pH 7.4 (BioBasic, Toronto, Canada) including Phosphatase and Protease inhibitors (Roche, Basel, Switzerland). After homogenization protein concentrations were determined using a bicinchoninic acid protein assay kit (Thermo Scientific, Rockford, IL, USA), and a Varioskan multimode plate reader (Thermo Scientific). Thirty micrograms of total protein were loaded to a Criterion XT precast gel (4–20% bis–tris) (Biorad, CA, USA). Proteins were subsequently transferred to a nitrocellulose membrane and incubated with anti-FMRpolyG 8FM and 9FM (1:10,000) and anti-glyceraldehyde 3-phosphate dehydrogenase (GAPDH) (Abcam, Cambridge, England. Ab8245; 1:10,00, mouse monoclonal) as internal loading control. The membrane was scanned with ChemiDoc™ XRS+ imager and the intensity of the bands of interest was quantified using Image Lab software (Biorad, CA, USA).

### Transfection

Human ovarian granulosa tumour cells (COV434) were seeded for 50–70% confluence 1 day prior to transfection. For a 24 wells plate, a mixture containing 0.5 μg DNA (Extracted according to manufacture orders, using NucleoBond Xtra Midi Plus kit, Macherey-Nagel, Germany) and 1.5 μl of Mirus (MirusBio LLC, WI, USA) at a final volume of 50 μl of Dulbecco’s Minimum Essential Medium (DMEM; Biological Industries) was used. Following incubation of 30 min, the mixture was added dropwise to each well. 72 h following transfection, the cells were permeabilized in 0.5% Triton X-100 in 4% PFA for 15 min and then fixed in 4% PFA for 20 min and washed with PBSx1.

Three plasmids used for transformation were kindly provided by Dr. Charlet-Berguerand at IGBMC, France. Briefly, 5′(99CGG)-GFP contains the 5′UTR of the FMR1 gene that contains 99 CGG repeats and was fused to eGFP sequence. Poly-glycine (FMRpolyG) was fused with FLAG protein (FMRpolyG-FLAG). In ATG (99CGG)-GFP, the weak ACG start codon was replaced for a strong ATG and no expression of FMRpolyG was enabled [16].

## Results

**Fig. 1:**
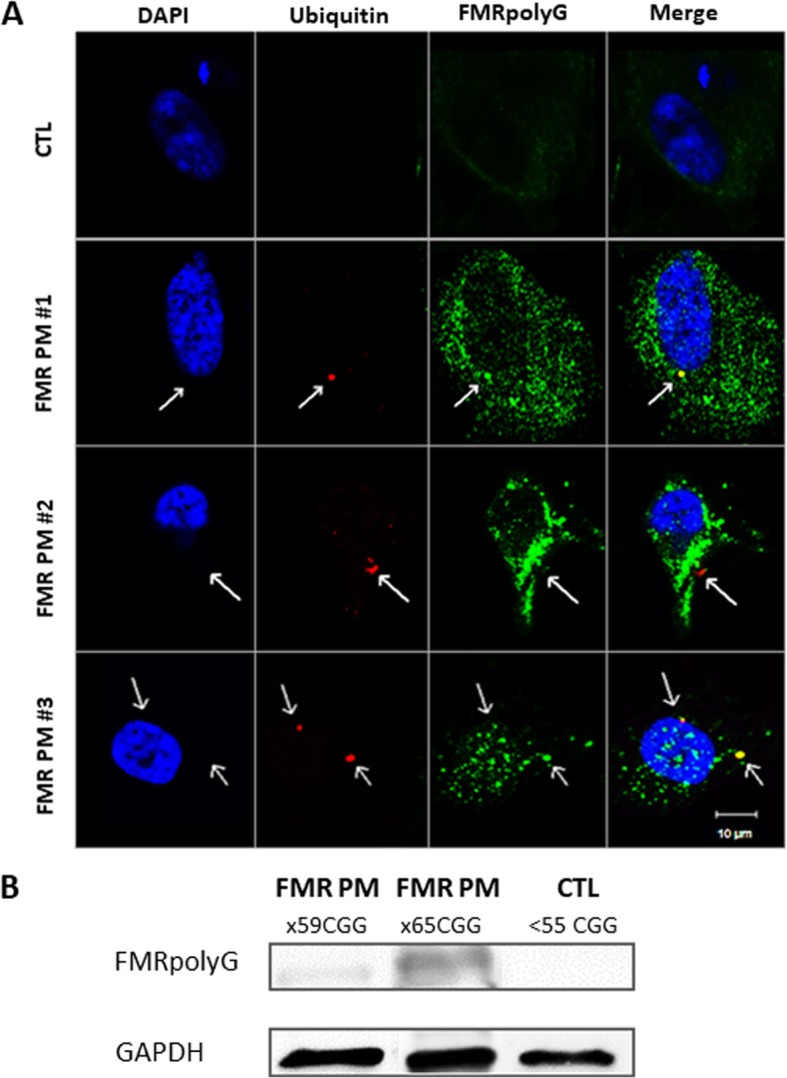
Granulosa cells from FMR1 premutation female carriers demonstrating co-localization of FMRpolyG and ubiquitin. **a** Fixated and stained granulosa cells from FMR1 premutation female carriers (FMR PM #1–3) displayed FMRpolyG expression and accumulation in ubiquitin-positive cytoplasmic inclusions. Granulosa cells from a non-carrier woman did not show any formation of FMRpolyG or ubiquitin inclusion bodies (CTL) Bar 10 μm. **b** Immunoblotting using anti-FMRpolyG revealed expression in granulosa cells of FMR1 premutation carriers (59 and 65 CGG repeats respectively) and none in granulosa cells from a non-carrier (CTL)

**Fig. 2:**
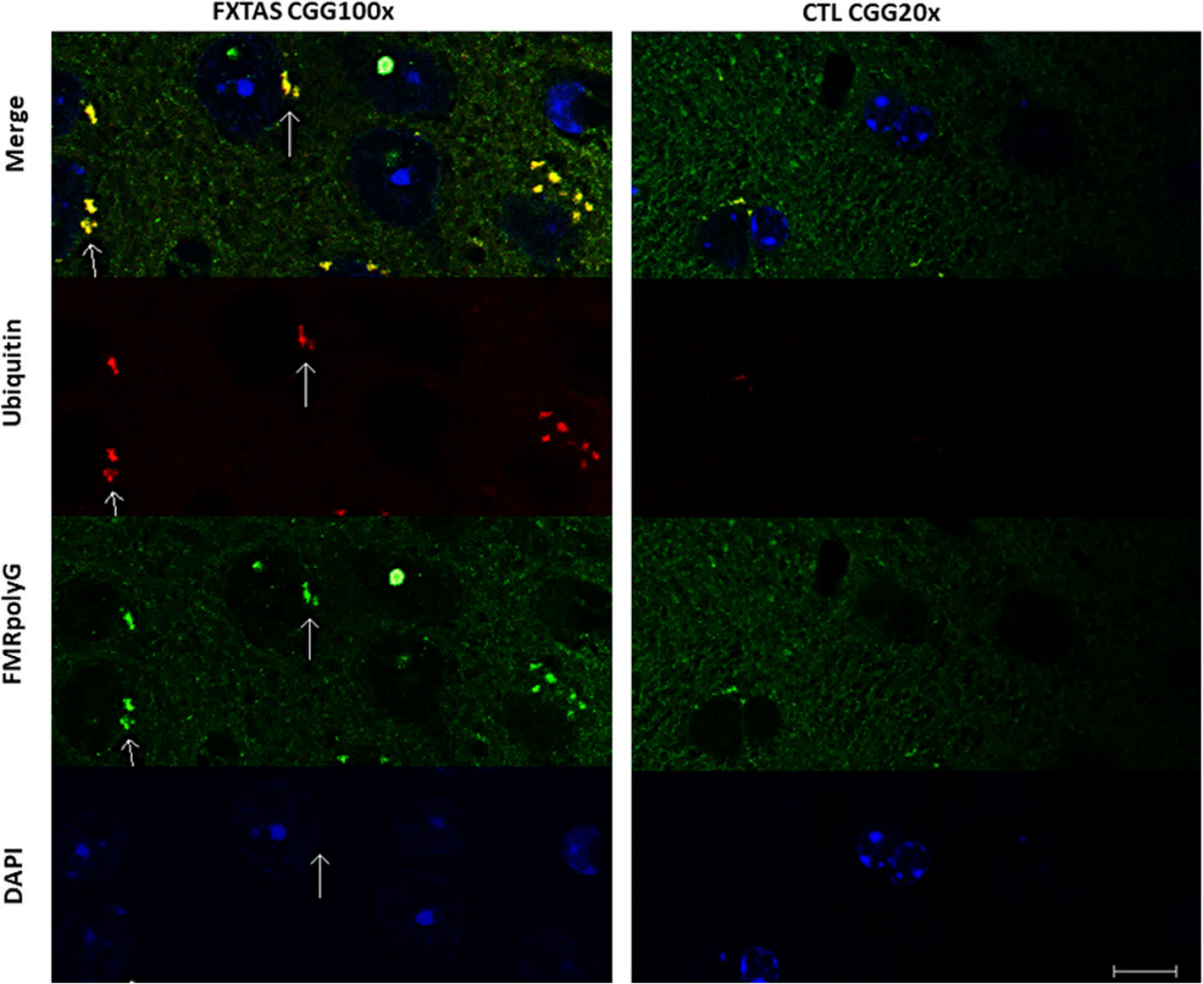
FMRpolyG accumulates in ubiquitin-positive inclusions in a mouse brain tissue with FXTAS. Paraffin embedded brains from a mouse disease model with 100 CGG repeats and a control mouse with 20 CGG repeats were sectioned and immune-stained. FXTAS mice brain tissue with 100 CGG repeats revealed co-localization of FMRpolyG and ubiquitin inclusions The control tissue with 20 CGG repeats showed no formation of FMRpolyG or ubiquitin inclusions. Bar 10 μm.

In the present study we demonstrated FMRpolyG expression in mural granulosa cells from all FMR1 premutation carriers. The FMRpolyG aggregates were detected mainly in cytoplasmic scattered aggregates in mural granulosa cells from FMR1 premutation carriers (Fig. 1a). In contrast to the perpetual co-localization of ubiquitin- and FMRpolyG-positive inclusions seen in our positive control slides with brain section of FXTAS transgenic mice (Fig. 2.), the intra-cytoplasmic FMRpolyG aggregates in mural granulosa cells were not consistently co-localized with ubiquitin within the individuals. Three FMR1 premutation carriers displayed co-localization of ubiquitin and FMRpolyG (Fig. 1a). However, in three additional carriers we found mainly intra-cytoplasmic FMRpolyG aggregates lacking the ubiquitin staining (Additional file 1S.). As predicted, we did not find FMRpolyG aggregates in woman with normal range number of CGG repeats (< 55 CGG repeats).

To evaluate quantified expression, we immunoblotted mural granulosa cells using anti-FMRpolyG and revealed no FMRpolyG expression in mural granulosa cells of a non-carrier woman. However, two FMR1 premutation carriers with 59 and 65 CGG repeats exhibited FMRpolyG expression. Notably, both FMR1 premutation carriers expressed FMRpolyG but exhibited different levels of expression (Fig. 1b).

**Fig. 3:**
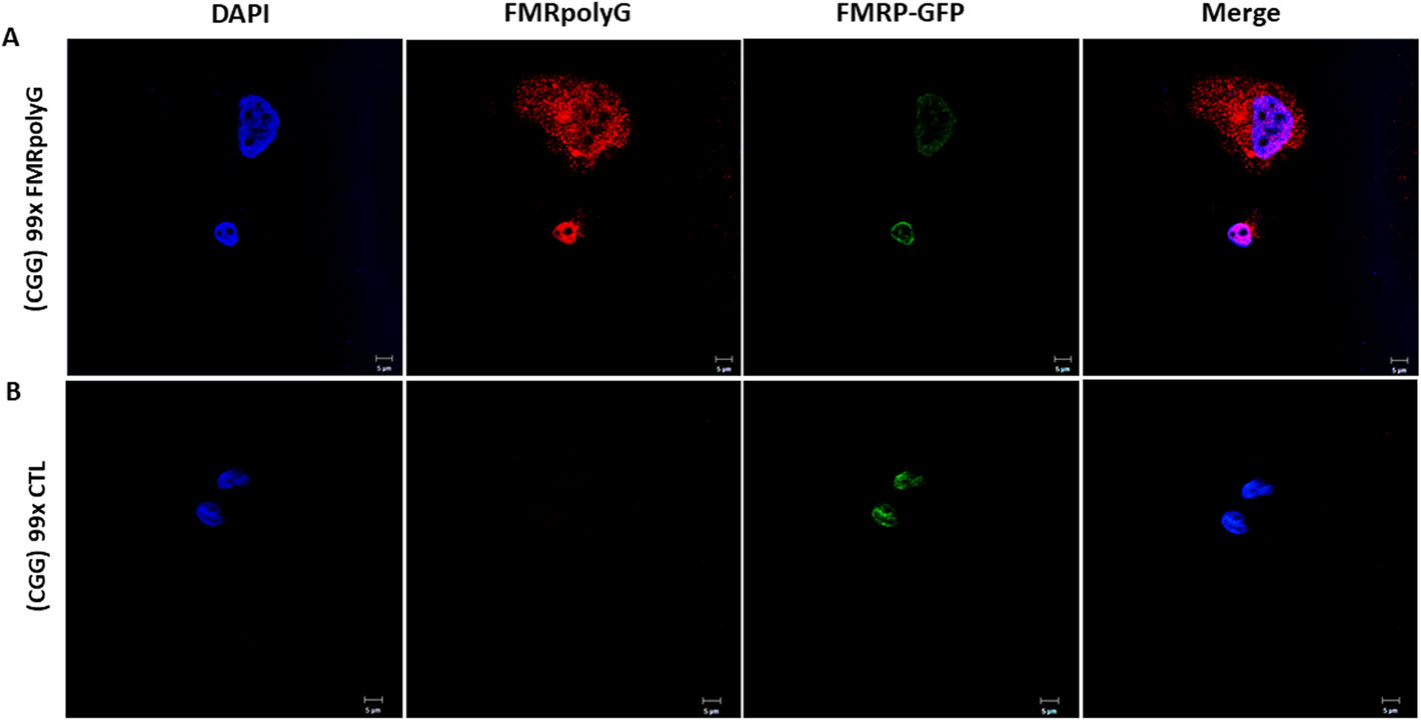
FMRpolyG aggragates in premutation transfected COV434 able to express the RAN translation product, FMRpolyG. **a.**FMRpolyG accumulation (Red) in premutation (× 99 CGG) ransfected COV434 expressing FMRpolyG. FMRP-GFP was detected in the nuclear of the transfected COV434 only (Green). Bar 10 μm. **b** Premutation (× 99 CGG) transfected COV434, unebaled to express FMRpolyG, did not exhibit FMRpolyG expression. Bar 20 μm

To validate that the transfected COV434 characteristics, related to RAN translation, resemble human granulosa cells of women undergoing IVF treatments, we stained premutation transfected COV434 (× 99 CGG repeats), with and without FMRpolyG expression, as described. To isolate the FMRpolyG staining from FMRP-GFP expression, immunostaining with Cy3 secondary antibody confirmed FMRpolyG expression only in the COV434 cells transfected with 99 CGG repeats expressing the FMRpolyG protein (Fig. 3a).

**Fig. 4:**
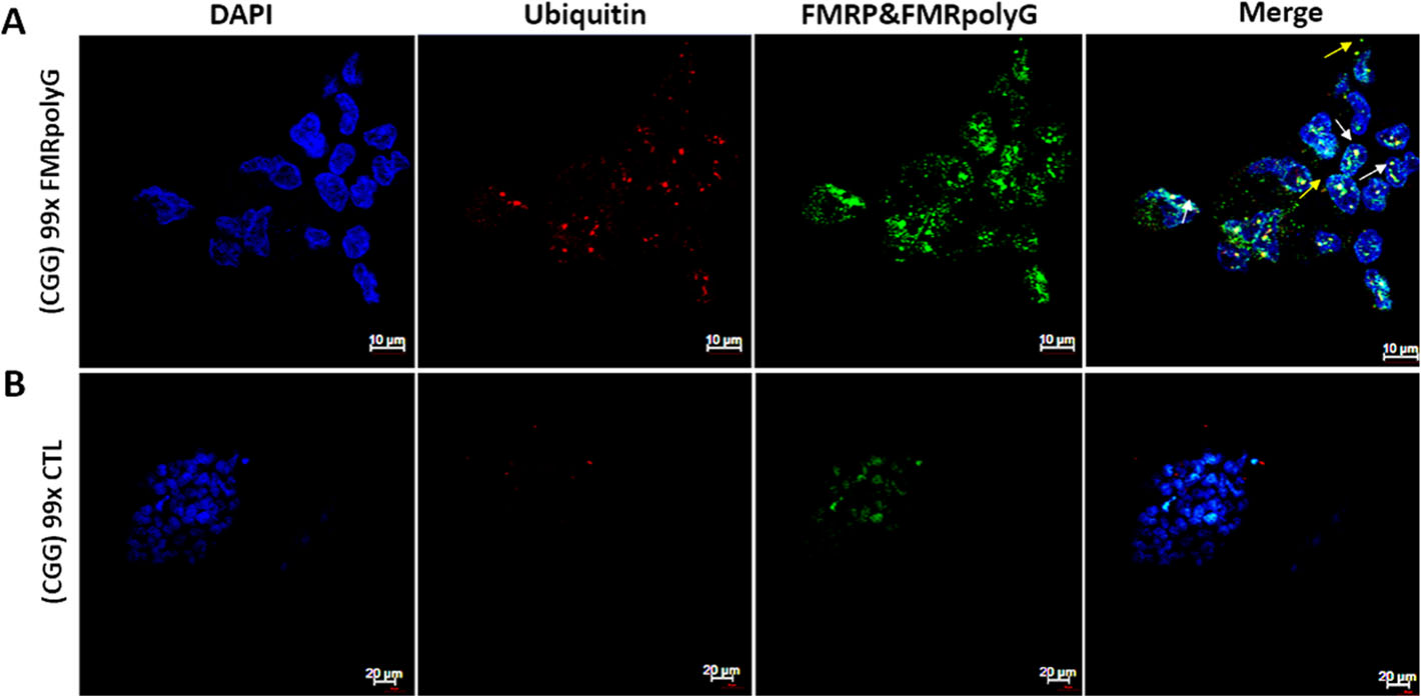
Transfected COV434 demonstrated positive co-staining ubiquitin and FMRpolyG aggregates**. a** Co-localization of FMRpolyG and ubiquitin in cytoplasmic and nuclear agreggates detected in premutation (× 99 CGG) ransfected COV434, expressing FMRpolyG. Bar 10 μm. **b** Premutation (× 99 CGG) transfected COV434, unebaled to express FMRpolyG, did not exhibit FMRpolyG accumulation. Bar 20 μm

We further explored ubiquitin activity in the transfected cells model. COV434 cells, transfected with 99 CGG repeats and expressing the FMRpolyG protein exhibited co-localization of ubiquitin- and FMRpolyG-positive staining in both nuclei and intra-cytoplasmic aggregates (Fig. 4a). COV434 cells, transfected with 99 CGG repeats but unable to express the FMRpolyG protein demonstrated only FMRP-GFP expression without FMRpolyG aggregates nor ubiquitin inclusions (Fig. 4b).

To confirm that the samples used in this study correlated with former reports on FMR1 elevation [29, 30], we used RT-qPCR analysis. Mural granulosa cells of FMR1 premutation carriers showed a significant (*p* = 0.02) elevation in *FMR1* mRNA transcript levels compared to non-carriers (1.56 ± 1.1 and 0.3 ± 0.2 folds respectively). However, in one FMR1 premutation carrier we have found decreased FMR1 mRNA expression level of 0.75 folds (Table 1).

**Table 1:** Characteristics of the control women and the FMR1 premutation carriers with FMRpolyG expression

	Woman’s age	No. of CGG repeats		No. of Oocyte retrieved	*FMR1* mRNA level (In mural granulosa cells)
		Allele 1	Allele 2		
CTL#1	36	< 45	< 45	16	0.29
CTL#2	27	< 45	< 45	21	0.29
CTL#3	30	< 45	< 45	10	0.68
CTL#4	36	< 45	< 45	3	0.47
CTL#5	35	< 45	< 45	8	0.11
FMR1 PM #1	28	64	< 45	20	3.98
FMR1 PM #2	25	75	< 45	37	2.04
FMR1 PM #3	26	123	< 45	3	1.39
FMR1 PM #4	37	82	< 45	10	0.75
FMR1 PM #5	28	72	< 45	10	1.17
FMR1 PM #6	31	65	< 45	11	1.72

## Discussion

This study demonstrates FMRpolyG accumulation in mural granulosa cells and co-localization with ubiquitin, thus suggesting that the RAN translation product, FMRpolyG, may play a role in the pathogenesis of FXPOI.

The mechanisms leading to FXPOI require further investigation. However, new advances in understanding the molecular mechanisms of FXTAS and other triplet neurological diseases have led researchers to believe that there are similarities in the pathophysiology between FXPOI and neurological diseases. Previous studies have demonstrated a cellular accumulation of FMR1 mRNA in both FXTAS [31] and FXPOI [32], that might lead to a toxic RNA gain-of-function mechanism. Recent studies suggested that non-canonical RAN translation [33] may play a role in the disease process and inclusions formation in FXTAS and other repeat expansion disorders. Todd et al. [24] revealed that the RAN translation product, FMRpolyG, is expressed and accumulated in ubiquitin-positive intranuclear and perinuclear inclusions in brain tissue from FXTAS patients but not controls. Buijsen et al. [26, 34] demonstrated the accumulation of intranuclear inclusion bodies of ubiquitin containing FMRpolyG in the nervous system and in various systemic organs from FXTAS as well as in stromal cells of an ovary from a FXPOI patient. Herein we aimed to explore whether FMRpolyG accumulates in folliculogenesis related cells, such as granulosa cells.

Mural granulosa cells from all FMR1 premutation carriers revealed an accumulation of FMRpolyG protein. In contrast to the large ubiquitin-positive inclusion body previously observed in the nervous system and in various systemic organs [24, 26], in our study FMRpolyG accumulates in the cytoplasm of granulosa cells and not in the nucleus. FMRpolyG accumulation and its co-localization with ubiquitin varied within the group of the FMR1 premutation carriers. The pattern of co-localization of FMRpolyG and ubiquitin in FXTAS involves a single double stained inclusion granule. In all three FMR1 permutation carriers with positive ubiquitin and FMRpolyG co-localization, we observed multiple small FMRpolyG granules, with little ubiquitin staining. Ubiquitin staining was found in some of the FMR1 permutation carriers in which a large portion of the FMRpolyG aggregates were scattered in the cytoplasm and did not co-localize with the positive ubiquitin staining.

FMRpolyG was not detected in the control group of non-carriers. A cloudy perinuclear staining was observed. Sellier et al. [16] performed a fusion of expanded CGG repeats in the glycine frame to FLAG and confirmed that FMRpolyG may be translated with short stretches of CGG repeats (x30CGG) in control individuals or even without any CGG repeat. This might be a result of short upstream open reading frames that are generally translated into small and usually undetectable peptides. However, when fused with large tags these peptides are more stable and detectable proteins which are then detected [35]. Moreover, they suggested that an expansion over 70 repeats is necessary for detection of FMRpolyG aggregates. This might explain the constitutive image we found in all non-carrier women.

Interestingly, we found a significant increase in FMR1 gene expression levels in FMR1 permutation carriers compared to non-carriers (Table 1.). Moreover, immunoblotting revealed higher FMRpolyG expression correlated with higher CGG repeats number (Fig. 1b). We suggest it would be noteworthy to further explore the correlation between the two mechanisms (RNA-gain-of-function and RAN translation) and identify the role of the intranuclear accumulation and the aberrant configuration of the *FMR1* mRNA in the process of RAN translation together with the expression of the FMRpolyG protein and other RAN translation products. We assume that the accumulation of *FMR1* mRNA in mural granulosa cells may be a contributing factor to the FMRpolyG expression.

Next, we compared clinical data of the patients in this study. Despite similar mean basal follicle stimulating hormone (FSH) and luteinizing hormone (LH) serum levels (Table 2.), FMR1 premutation carriers required enhanced stimulation for them to produce oocytes during the IVF procedure (Table 2.). Peak Estradiol levels at the day of human chorionic gonadotropin (hCG) were higher in the non-carriers group compared to the levels in the FMR1 premutation group (Table 2.). Despite two FMR1 premutation carriers with good response to COH with a high number of oocytes retrieved (Table 1.), the other four FMR1 premutation carriers participated in this study demonstrate an ongoing deterioration of ovarian functionality despite a young age. Even after retrieving many oocytes the pregnancy rate was lower compared to non-carrier women (Table 2.) insinuating more mechanisms are involved and might impact following processes.

**Table 2:** Study groups’ fertility features (median and ranges)

	Non-carriers	FMR1 premutation carriers	*p*-Value
Age (yrs)	36 (28–37)	29 (26–36)	0.2
Parity	0 (0–5)	0 (0–1)	0.3
Basal FSH (IU/L)	6.1 (4.3–7)	7.1 (3.4–7.9)	0.5
Basal LH (IU/L)	4.0 (2.3–7)	5.3 (2.7–5.9)	0.4
Basal FSH/LH ratio	1.5	1.3	
Total Gonadotrophins used for stimulation (IU)	2200 (1300–5350)	2950 (1875–6000)	1.8 × 10^9^
Peak of Estradiol- pmole/L	8113 (2386–8127)	3736 (1586–9266)	0.2
No. of oocytes retrieved	10 (3–21)	9 (1–37)	0.6
No. of pregnancies	0.5 (0–5)	0 (0–1)	0.2
FMR1 repeats (Range)	< 55	64–123	

Our findings cannot explain the variations in FMRpolyG aggregates formation in FXTAS and FXPOI and further investigation is essential. We postulate that FMRpolyG accumulates mainly in the cytoplasm and not in the nucleus due to several reasons. First, mural granulosa cells and brain cells have different characterizations and functions. Granulosa cells differentiate proximately to ovulation whereas brain cells are localized and function in the brain tissue for a longer time. Previous studies demonstrated ubiquitin-positive FMRpolyG containing an intranuclear inclusion in non-dividing or slow dividing cells, such as glomeruli, distal tubule of the kidney, zona glomerulosa and zona reticularis of adrenal gland, cardiomyocytes and various neuronal cells [34, 36]. While, in our study FMRpolyG accumulated mainly in the cytoplasm of granulosa cells, representing rapidly dividing cells. Studies on rapidly dividing cells showed higher proteasome activity, disposing toxic proteins during cell division [37]. Whereas, compared to cancer cells, in slowly growing cells proteasome subunits have been downregulated [38]. This might explain the scattered pattern of FMRpolyG aggregates in granulosa cells compared to single inclusions found in brain tissue of FXTAS patients. Moreover, the process of nuclear aggregates of FMRpolyG accumulation is an ongoing process [8]. We suggest to further explore whether the proteasome complex dispose the accumulating FMRpolyG throughout cell division. Furthermore, in our study all the patients were females, while in previous reports, presenting brain tissues, all patients were male FMR1 premutation carriers. The X inactivation process occurring in female FMR1 premutation carriers might in some way attenuate the process of FMR1 transcription causing this difference in localization. We presume that other variables such as the CGG repeats tract length and the pathophysiology of each female FMR1 premutation carrier might explain the variability in the co-localization of the FMRpolyG. We did not find any correlation between the FMRpolyG accumulation and the FMR1 premutation carrier’s ovarian dysfunction. Additional studies are needed to better understand this phenomenon.

There are several known mechanisms involved in both male and female FMR1 premutation carriers. The association between the FMR1 mRNA accumulation and the RAN translation mechanisms is still obscure and requires further investigation. Furthermore, FMRpolyG accumulation was previously demonstrated in ovary stromal cells but not in cells involved with folliclogenesis [26]. The establishment of a well representative cell line disease model is significant to further explore the mentioned mechanisms in FXPOI and to examine whether the FMR1 premutation carriers’ pathogenesis is indeed related to the premutation state. Our next goal is to characterize the fertility phenotype and cell maintenance features of the transfected COV434 models and compare them to mural granulosa cells. Nonetheless, we suggest that the transfected COV434, with and without FMRpolyG expression, may enable isolating the effect of the RNA-gain-of-function mechanism from RAN translation and shed a light on the function of each mechanism in FXPOI pathogenesis. Recent findings suggesting common toxic pathways between FXPOI and FXTAS are rather exciting. We believe that the cytotoxicity of neurons in FXTAS and of granulosa cells in FXPOI is due to similar cellular mechanisms. More studies are necessary to fully understand the toxic role of these mechanisms in FXPOI.

## Conclusions

In summary, we detected FMRpolyG accumulation in mural granulosa cells of FMR1 premutation carriers. We revealed a different pattern of FMRpolyG formation with multiple cytoplasmic and nuclear aggregates in FXPOI compared to a single inclusion in FXTAS, which we could not yet explain. It is imperative that we further characterize and examine whether the premutation transfected COV434 can offer a suitable disease model. Further investigation is needed to understand the pathological effect of FMRpolyG expression and elucidate the molecular basis of cellular toxicity in females FMR1 premutation carriers. Understanding the mechanisms resulting in the pathological phenotype might significantly contribute and assist physicians in tailoring suitable treatment protocol and fertility preservation to FMR1 premutation carriers.

## Supplementary information

**Additional file 1S.** Mural granulosa cells of FMR1 premutation carriers exhibited FMRpolyG accumulation without ubiquitin inclusions. Two additional FMR1 premutation carriers demontrated FMRpolyG positive staining withoout co-localization with ubiquitin (FMR PM #4–6). Granulosa cells of a non-carrier did not displayed aggregates formation (CTL#3). Bar 10 μm.

**Additional file 2S.** AMH expression in cultured mural granulosa cells. Immunostaining of mural granulosa cells demonstrated AMH expression in both FMR1 premutation carrier (FMR PM) as non-carrier (CTL). Bar 20 μm.

## Data Availability

The datasets used and/or analyzed during the current study are available from the corresponding author on reasonable request.

## Abbreviations

AMH: Anti Müllerian hormone
BSA: Bovine serum albumin
cDNA: Complementary deoxyribonucleic acid
COH: Controlled ovarian hyperstimulation
DGCR8: DiGeorge critical region 8
DMEM: Dulbecco’s Minimum Essential Medium
DMPK: Dystrophia myotonica protein kinase
FMR1: Fragile X mental retardation 1
FSH: Follicle stimulating hormone
FXPOI: Fragile X-Associated Premature Ovarian Insufficiency
FXS: Fragile X Syndrome
FXTAS: Fragile X-associated tremor/ataxia syndrome
GAPDH: Glyceraldehyde 3-phosphate dehydrogenase
hCG: Human chorionic gonadotropin
IVF: In vitro fertilization
LH: Luteinizing hormone
OPU: Ovum pick-up
PGD: Pre-implantation genetic diagnosis
RAN translation: Repeat associated non-AUG initiated translation
