# Supplementary figures and images for: FMRpolyG accumulates in FMR1 premutation granulosa cells
Source: J Ovarian Res. 2020 Feb 26;13:22. doi: 10.1186/s13048-020-00623-w (PMC7045455; doi:10.1186/s13048-020-00623-w)

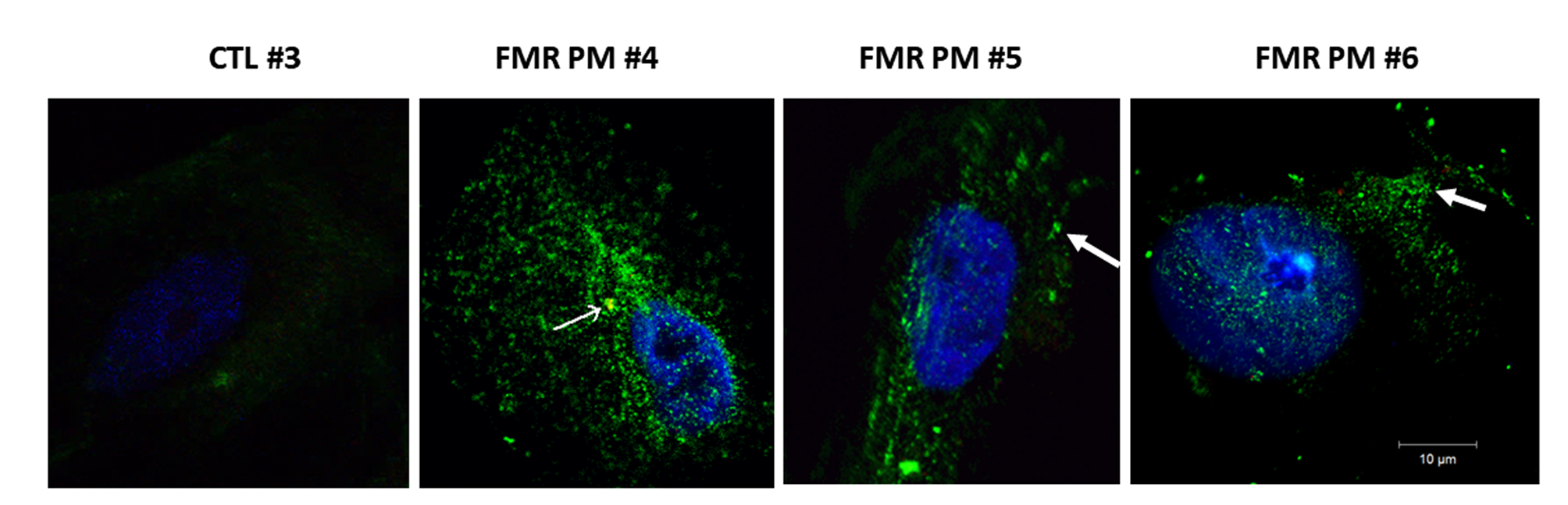

Supplement: Supplementary file 1 — Additional file 1S. Mural granulosa cells of FMR1 premutation carriers exhibited FMRpolyG accumulation without ubiquitin inclusions. Two additional FMR1 premutation carriers demontrated FMRpolyG positive staining withoout co-localization with ubiquitin (FMR PM #4–6). Granulosa cells of a non-carrier did not displayed aggregates formation (CTL#3). Bar 10 μm. [file 13048_2020_623_MOESM1_ESM.tif]

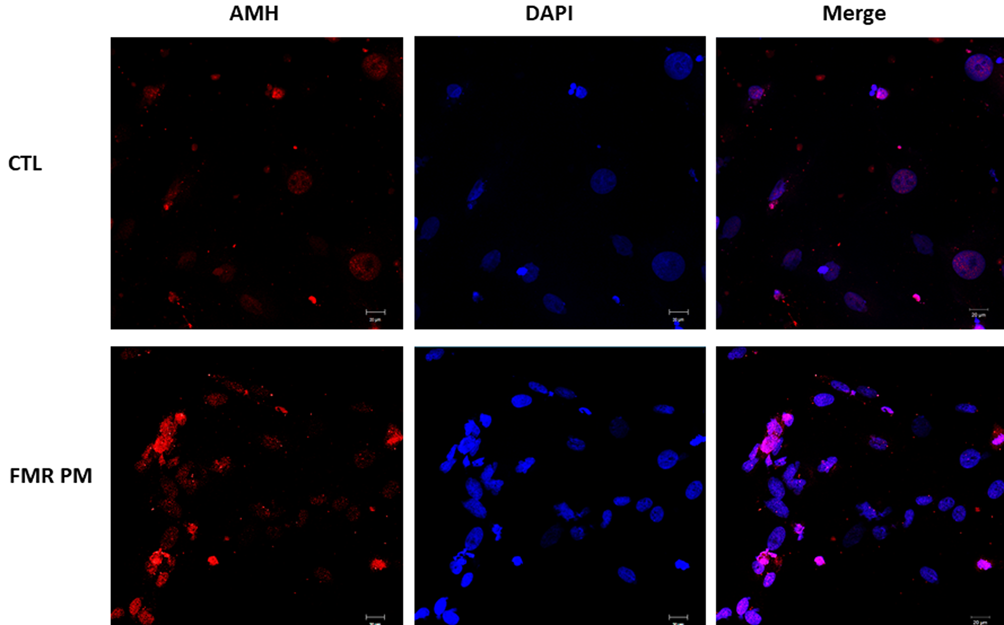

Supplement: Supplementary file 2 — Additional file 2S. AMH expression in cultured mural granulosa cells. Immunostaining of mural granulosa cells demonstrated AMH expression in both FMR1 premutation carrier (FMR PM) as non-carrier (CTL). Bar 20 μm. [file 13048_2020_623_MOESM2_ESM.tif]
